# Cardiac leptin overexpression in the context of acute MI and reperfusion potentiates myocardial remodeling and left ventricular dysfunction

**DOI:** 10.1371/journal.pone.0203902

**Published:** 2018-10-12

**Authors:** David Kain, Amos J. Simon, Avraham Greenberg, Danny Ben Zvi, Boris Gilburd, Jacob Schneiderman

**Affiliations:** 1 Department of Neurobiology, Tel Aviv University, Tel Aviv, Israel; 2 Cancer Research and Institute of Hematology, Sheba Medical Center, Sackler Faculty of Medicine, Tel Aviv University, Israel; 3 Department of Developmental Biology and Cancer Research, The institute for Medical Research Israel-Canada, The Hebrew University-Hadassah medical School, Jerusalem, Israel; 4 Center for Autoimmune Diseases, Sheba Medical Center, Sackler Faculty of Medicine, Tel Aviv University, Israel; 5 Department of Vascular Surgery, Sheba Medical Center, Sackler Faculty of Medicine, Tel Aviv University, Isreal; University of Cincinnati College of Medicine, UNITED STATES

## Abstract

**Background:**

Acute MI induces leptin expression in the heart, however the role of myocardial leptin in cardiac ischemia and reperfusion (IR) remains unknown. To shed light on the effects of elevated levels of leptin in the myocardium, we overexpressed cardiac leptin and assessed local remodeling and myocardial function in this context.

**Methods and results:**

Cardiac leptin overexpression was stimulated in mice undergoing IR by a single intraperitoneal injection of leptin antagonist (LepA). All mice exhibited a normal pattern of body weight gain. A rapid, long-term upregulation of leptin mRNA was demonstrated in the heart, adipose, and liver tissues in IR/LepA-treated mice. Overexpressed cardiac leptin mRNA extended beyond postoperative day (POD) 30. Plasma leptin peaked 7.5 hours postoperatively, especially in IR/LepA-treated mice, subsiding to normal levels by 24 hours. On POD-30 IR/LepA-treated mice demonstrated cardiomyocyte hypertrophy and perivascular fibrosis compared to IR/saline controls. Echocardiography on POD-30 demonstrated eccentric hypertrophy and systolic dysfunction in IR/LepA. We recorded reductions in Ejection Fraction (p<0.001), Fraction Shortening (p<0.01), and Endocardial Fraction Area Change (p<0.01), and an increase in Endocardial Area Change (p<0.01). Myocardial remodeling in the context of IR and cardiac leptin overexpression was associated with increased cardiac TGFβ ligand expression, activated Smad2, and downregulation of STAT3 activity.

**Conclusions:**

Cardiac IR coinciding with increased myocardial leptin synthesis promotes cardiomyocyte hypertrophy and fibrosis and potentiates myocardial dysfunction. Plasma leptin levels do not reflect cardiac leptin synthesis, and may not predict leptin-related cardiovascular morbidity. Targeting cardiac leptin is a potential treatment for cardiac IR damage.

## Introduction

Heart failure (HF) is a growing health problem worldwide, affecting at least 10% of all patients suffering from heart disease. There are approximately 790,000 new cases of acute myocardial infraction (MI) in the US every year, with HF therapy related expenditure exceeding $40 billion dollars[1,2].

Patients surviving acute MI exhibit variable degrees of HF. Post-MI ventricular dysfunction correlates with the extent of myocardial ischemic injury[3]. However, the most frequently used therapeutic modality for coronary revascularization via primary percutaneous coronary intervention (PPCI) may exert additional damage to the ailing myocardium and extend the infarct size. This response is related to reoxygenation processes in ischemic cardiomyocytes, which are more pronounced in case of delayed reperfusion. Myocardial IR injury activates the renin-angiotensin aldosterone system (RAAS), resulting in released angiotensin II (AngII) and endothelin-1 (ET-1), both of which drive myocardial remodeling via leptin induction and mediation[4].

The leptin hormone affects multiple systemic functions, such as energy expenditure, hematopoiesis, immune response, and angiogenesis[5]. Leptin plasma levels reflect its synthesis by the adipose cell mass throughout the body, which is the primary source of the hormone. Furthermore, in addition to its endocrine role, leptin is induced in cardiovascular organs by vascular SMCs, fibroblasts, and infiltrating macrophages, and drives localized tissue remodeling *via* paracrine and autocrine pathways. Local leptin expression promotes local oxidative stress, attracts macrophages and lymphocytes, induces pro-inflammatory cytokines, and activates metalloproteinases[6]. In the clinical setting, multiple deleterious effects of leptin have been suggested, such as promoting the generation of unstable atherosclerotic plaques in the carotid artery[7] and driving the expansion of aortic aneurysms[8]. Nevertheless, in the context of myocardial IR it remains unclear to what extent *in situ* cardiac leptin affects remodeling and subsequent myocardial function. Studies in mice have demonstrated that leptin regulates cardiac oxidation of glucose and fatty acids, thereby providing protection against cardiac lipotoxicity[9]. Several murine models and *ex vivo* analyses revealed that leptin has cardioprotective properties in myocardial ischemia[10–13]. In contrast, clinical data suggest that plasma leptin levels correlate with cardiovascular morbidity, such as in obesity, acute MI, HF, and stroke[14–16]. Moreover, hyperleptinemia is often considered a surrogate marker for cardiovascular disease[17]. Rats that were subjected to acute MI demonstrated preservation of myocardial function when cardiac leptin activity was counteracted[18,19]. To reconcile seemingly conflicting observations, we hypothesized that although cardiac leptin is an integral part of the normal protective cardiac response to stress, hyper-activation of cardiac leptin signaling may potentiate post-MI myocardial remodeling, leading to more extensive damage and increased heart failure.

Plasma leptin is elevated in patients suffering from chronic HF[20] and acute MI[21]. However, blood leptin level in the context of myocardial IR may not necessarily reflect cardiac leptin expression. As human cardiac samples during acute MI are unobtainable, leptin transcript levels in LV cardiomyocytes have not been determined in this setting. The current study utilizes wild type mice to determine the relationship between augmented levels of *in situ* cardiac leptin coinciding myocardial IR, and examines plasma leptin, post-MI cardiac remodeling, and functional damage. Here we demonstrate that long-term overexpression of cardiac leptin is associated with potentiated post-MI HF, without necessarily promoting hyperleptinemia.

## Methods

**Fig 1:**
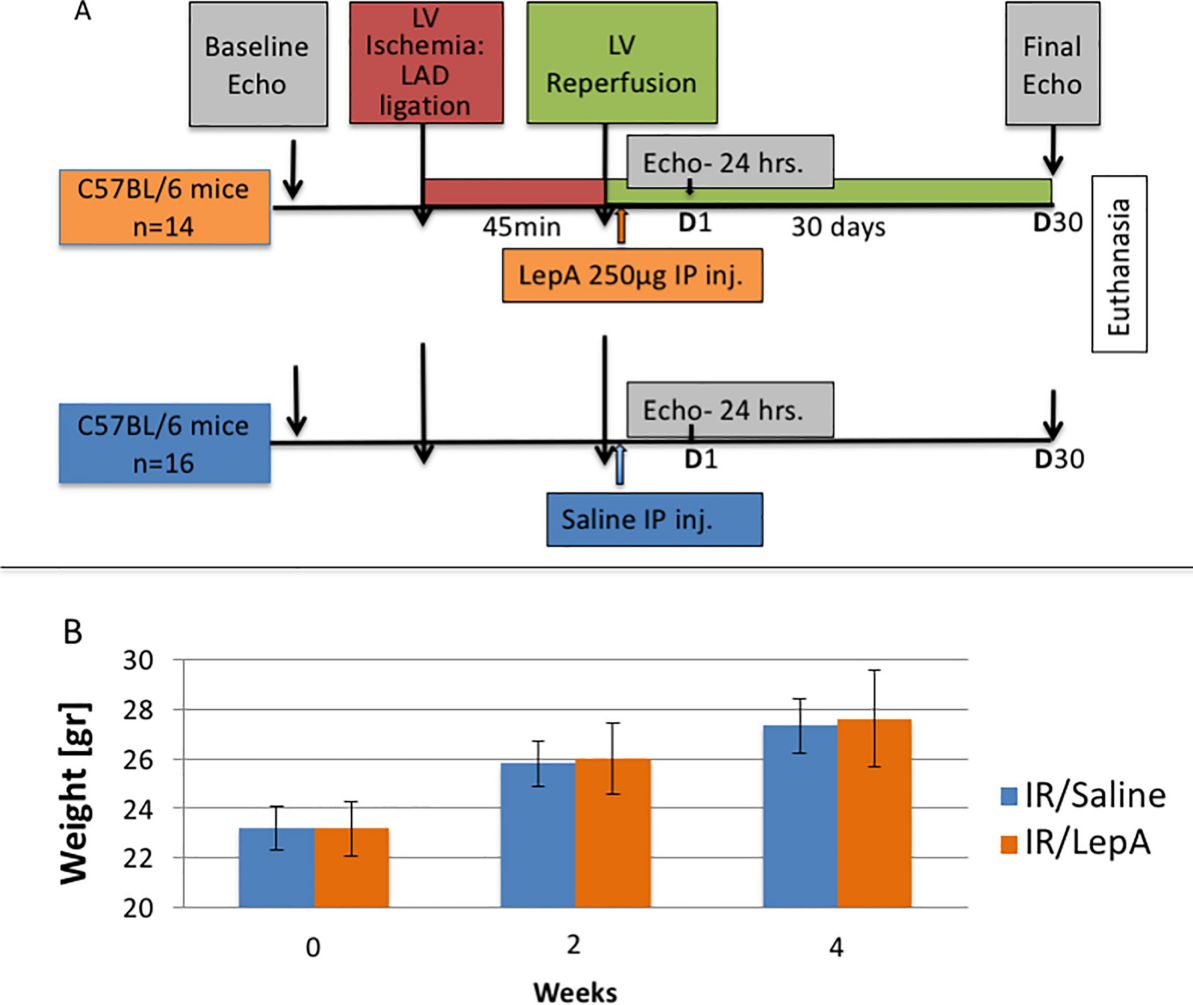
Study design. (A) Mice in both groups underwent ischemic injury by LAD ligation, which lasted 45 minutes, and received a single injection of either LepA 250μg in 200μl saline solution or 200μl saline alone at reperfusion. Echocardiography was performed at baseline, 24 hours and 30 days postoperatively; (B) Body weight (mean±SE) time course analysis.

The experimental model (Fig 1A): A murine model of increased cardiac leptin expression in the context of post-MI and reperfusion injury.

Experiments were performed in compliance with animal welfare regulations of the authors’ institutions conforming to the “Position of the American Heart Association on Research Animal Use,” adopted by the American Heart Association on November 11, 1984. All experiments involved C57BL6/J male mice, age 11–12 weeks (22–25 gr). Mice were purchased from ENVIGO RMS (ISRAEL) LTD, Jerusalem. All animals were maintained in micro-isolator cages under specific pathogen-free conditions in a room with a temperature of 24°C, at relative humidity of 55–65%, and under a 12-h light–dark cycle. Mice underwent baseline echocardiography and myocardial IR was induced. Briefly, mice were positioned supine and stretched over a warming pad at 37°C. After induction with 2.5–3% isoflurane, the mice were intubated and ventilation anesthesia was maintained with 1.5–2% isoflurane (miniVent, Harvard apparatus). Left thoracotomy was performed through the 3^rd^ intercostal space to allow exposure of the heart. The proximal left anterior descending (LAD) coronary artery was ligated using a nylon 8–0 suture, which was applied around the vessel 1 mm beyond the edge of the left atrial appendage. Segmental ischemia in the left ventricle was verified by visual inspection (i.e., an area of myocardium turning pale after tying the suture). Chest wall and the skin were closed with a running 5–0 silk suture, mice were weaned off the respirator and rapidly regained consciousness. After 45 min of cardiac ischemia, each mouse was re-anesthesized, chest wall was reopened, and the LAD occluding suture removed. Reperfusion was visualized by the appearance of local hyperhemia in the left ventricular area, which was previously ischemic and pale. 250μg of leptin antagonist (LepA)[22] in 200μl saline solution, or 200μl saline solution without LepA were instantly injected intraperitoneally (IP) at reperfusion. The chest wall was then carefully closed and 5–0 silk suture was used for the skin. Mice in this experiment were followed up for 30 days. Mice were euthanized by CO_2_ overdose.

Echocardiography was performed under light anesthesia at 24 hours and 30 days after IR. Echocardiographic data included short, and long axis views, and also m-mode recording. Mouse weight was assessed prior to surgery, 2 weeks, and 30 days PO. To induce endogenous cardiac leptin overexpression we used a strategy as suggested in a previously published study[23], and briefly disrupted the leptin/leptin receptor pathway by a single intraperitoneal (IP) injected leptin antagonist (LepA) solution at a dose of 250μg.

### Mice

A series of 34 mice underwent left thoracotomy and ligation of the LAD lasting 45 min. At coronary reperfusion each mouse received an IP injection of either LepA or saline. Four mice were excluded from the study, one died from anesthetic mishap, and 3 were disqualified after they were found to escape myocardial ischemic injury because of premature loosening of the LAD ligature (as viewed by inspection and confirmed histologically). Thus, 30 mice were treated by IR/LepA (n = 14), or IR/saline (n = 16), and were followed up for 30 days. Additional 21 mice undergoing a similar procedure were euthanized at designated intervals to provide for a time course analysis of tissue mRNA expression and plasma leptin levels. Another group of 16 mice received a single IP injection of LepA or saline, that was not preceded by IR.

### Echocardiography

Echocardiography was performed to assess cardiac morphology and function using a commercially available mouse echocardiography system (Vevo 2100,VisualSonics, Toronto, Canada), equipped with a 30 MHz phased array. After a baseline echocardiography, all mice underwent IR with either LepA or saline IP injection at reperfusion. Follow up echocardiography was performed under general anesthesia 24 hours and 30 days postoperatively, using 1.5% isoflurane applied through a face mask. ECG was monitored to verify that echocardiography examinations are performed at heart rate of 400–450 bpm. A transthoracic two-dimensional mode was used for parasternal long- and short-axis views, through which the m-mode cursor was positioned perpendicular to the left ventricular (LV) septum and posterior wall, at the level of the papillary muscles. All echocardiograpy examinations were performed by an experienced technician blinded to the treatment groups, and all measurements were averaged for three consecutive cardiac cycles.

### Quantitative real-time PCR

RNA was prepared from frozen tissues using Trizol and converted to cDNA. Real-time qPCR was performed using ABI STEP ONE Detection System (Applied Biosystems) and Universal PCR Master Mix (Applied Biosystems) according to the manufacturer’s instructions. The TaqMan probes and primers for mouse leptin (assay ID number: Mm00434759), leptin receptor (assay ID number: Mm00440181_m1), NPPA (ANP) (assay ID number: Mm01255747), SMAD2 (assay ID number: Mm00487530), TGF-beta 1 (assay ID number: Mm01178820) were 'assay-on-demand' gene expression products (Applied Biosystems). The 0 hours samples served as the controls thus normalized to 1 in the analysis. The endogenous reference gene control was mouse GAPDH (assay ID number: Mm99999915).The results presented are fold changes based on the differences of normalized Ct values compared to control samples, assuming optimal primer efficiency (ΔΔCt). Results were analyzed using SDS 2.4 (Applied Biosystems) and Excel (Microsoft Corp) software. Each point assayed in triplicates, and triplicates were used for error calculation. 2 tail t-test was performed to calculate the statistical significance (p-values). In all analyses of mRNA expression for the various genes each time point represented a single mouse. Parallel assays on RNA samples from different mice subjected to the same conditions yielded similar pattern of results.

### Histochemistry and Immunohistochemistry

Formalin-fixed 5μm paraffin slides were stained with Masson trichrome, and assessed for cardiomyocyte hypertrophy. Slides from each treatment group (8 per group) were inspected with high magnification light microscopy (x400). Each slide was examined in at least 5 viewing fields of cardiomyocyte cross section to measure cardiac muscle cell area (μm^2^). To assess perivascular fibrosis (pvf), a similar number of slides were examined, focusing on areas beyond the margins of the infarct. 10 viewing fields were inspected under medium (x100) and high magnification (x400) for each slide. Positive nuclei expressing Psmad2 antigen as well as unstained nuclei were counted in 5 different slides from each treatment group (3 viewing fields per slide). Angiogenesis (VWF) was assessed in 5 slides in each group of treatment.

Formalin-fixed hearts collected from PO day 30 IR/LepA and IRI/saline treated mice were dehydrated, embedded in paraffin, and sectioned at 5 μm. Slides were warmed up to 60°C for 1 hour, de-waxed in xylene and rehydrated. The following immunohistochemical (IHC) stainings were done: for EC staining, Rabbit anti Human VWF Abs (Dako #A082), 1:250, pretreatment with Proteinase K (Invitrogen# 25530–015) for 20 min at RT and overnight incubation at 4°C; for Mac2, anti-mouse/human Mac2 (Galectin-3), Cedarlane #CL8942AP, Pretreatment -Target Retrieval Sol. (Dako S1699) heated up to 95C, cooled down to RT. overnight incubation at 4C; For mouse leptin, abcam 16227, 1:50; for mouse P-Smad2, abcam, ab3849, 1:3000, 30 minute citrate retrieval, 30 minute antibody incubation. Mouse p-STAT3, abcam ab76315, 1:20, 20 min EDTA retrieval, 30 min antibody incubation. Stained sections were reviewed using light microscopy. All analyses of histological data were performed by two experienced investigator blinded to the treatment groups.

### Leptin antigen level assessment

A Mouse/Rat Leptin Quantikine ELISA Kit MOB00: R&D Systems was used. Leptin antigen level was determined in mouse plasma samples according to the manufacturer instructions. Noteworthy, this kit does not distinguish between leptin and the LepA mutant (LepA).

### Statistics

2-tailed student’s t-test was used to compare IR/LepA treated mice with IR/Saline treated mice. Bars represent means, and error bars standard error of the mean. The number of measurements is indicated in each related figure legend. For qPCR data, error bars represent standard error of the mean of technical triplicates of a single measurement. However, qPCR analyses were repeated for different sets of tissue samples, yielding similar results as presented. One-sided Fisher's Exact test was used for comparative analysis of categorical outcome in LV mass analysis. The data was analyzed using the SAS ® version 9.3 (SAS Institute, Cary North Carolina).

## Results

Thirty wild-type mice (C57BL/6) survived the full 30-day experiment (Fig 1A).

All mice underwent LAD ligation to induce left ventricular myocardial ischemia, which lasted 45 minutes. Coronary reperfusion was followed by an immediate IP injection of LepA (IR/LepA, n = 14), or saline solution (IR/saline, n = 16). A single dose of LepA was injected IP to enhance endogenous leptin, specifically in the myocardium. A transient disruption of leptin / leptin receptor pathways did not cross physiological boundaries, causing neither detectable morbidity, nor evidence of systemic hormonal perturbations. All mice from both groups exhibited normal behavior and a similar pattern of weight gain throughout the experiment (Fig 1B).

### Post-MI cardiac dysfunction is augmented by myocardial leptin overexpression

**Fig 2:**
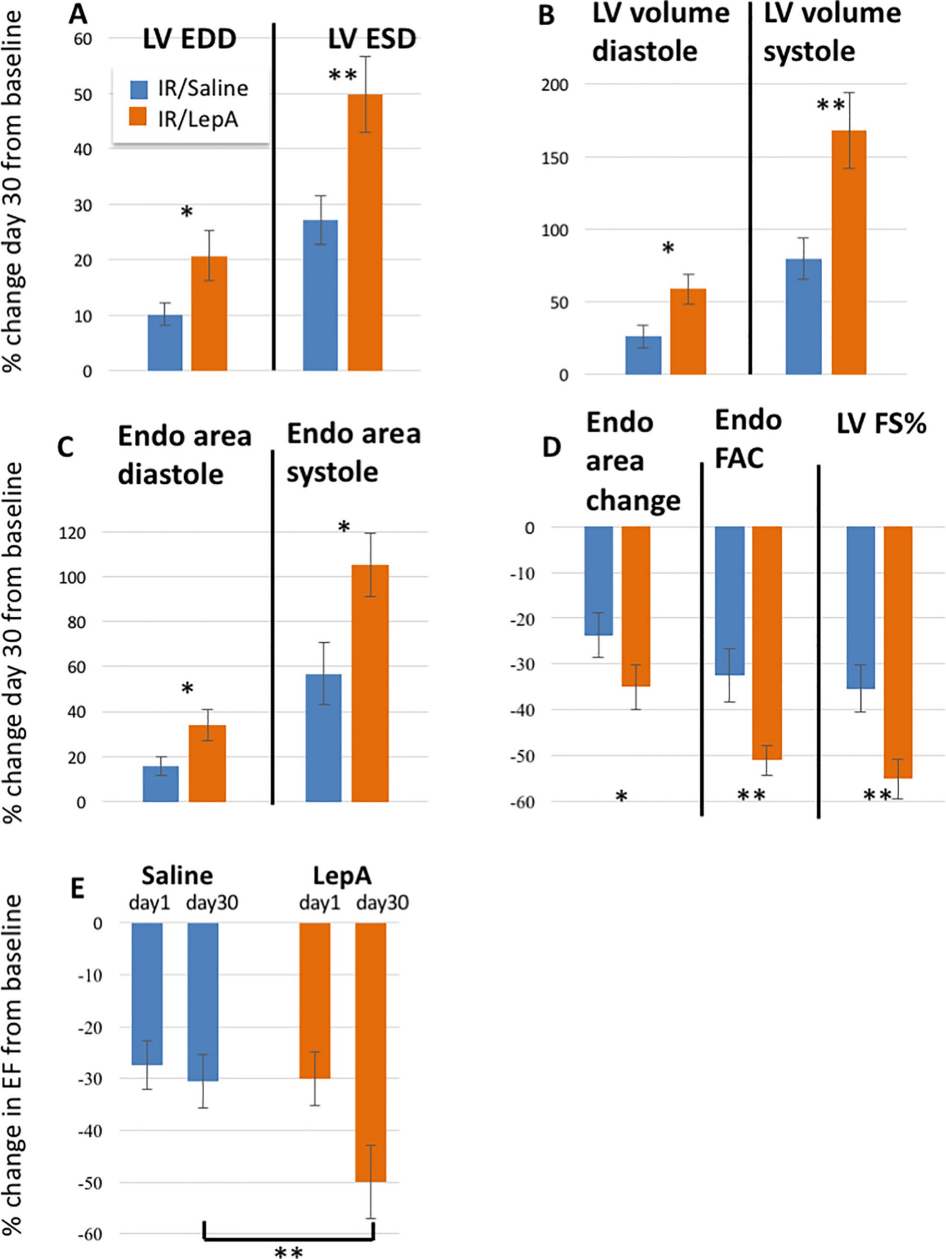
Echocardiographic analysis of multiple morphological and functional parameters in IR/LepA and IR/saline treated mice. (A-E): Echocardiography was performed at baseline, 24 hours and 30 days postoperatively. A: Increase in left ventricle (LV) end-diastolic diameter (EDD) and end-systolic diameter (ESD); B. Increase in LV volume at diastole and systole; C. Increase in endocardial (Endo) area in diastole and systole; D. Endo area change, Endo fractional area change (FAC)%, and LV fractional shortening (FS)%; E. Ejection fraction (EF)% on day1 and day 30. Values show average percent of change ± SE from baseline measurements. In all figures orange bar depicts IR/LepA treated mice, and blue bars IR/Saline treated mice. * p<0.05, ** p<0.01 by two-tailed unpaired student’s t-test between the two groups. Error bars indicate standard error. (data collected from 14 IR/saline and 16 IR/LepA treated mice).

Echocardiography was performed at baseline, 24 hours, and on POD-30 in IR/LepA and IR/saline treated mice. Cardiac morphology and function assessment was based on multiple echocardiographic parameters (Fig 2).

Echocardiography analyses revealed the development of eccentric hypertrophy, including dilation and significant reduction in LV function in IR/LepA versus (vs) IR/saline treated mice (Fig 2A–2E). In detail, echocardiography revealed increased LV end-diastolic dimension (LVEDD) (p<0.05); LV end-systolic dimension (LVESD) (p<0.01), (day 30 vs baseline), LV volume diastolic (p<0.05); LV volume systolic (p<0.01), (day 30 vs baseline). Jointly, these findings indicate significant LV enlargement, markedly affecting LV systolic function. These included increased endo area diastolic (p<0.05) and systolic (p<0.05), (day 30 vs baseline), and endocardial area change (p<0.05), (day 30 vs baseline). FAC% (p<0.01), (day 30 vs baseline), (p<0.05), (day 30 vs day 1).,FS% (p<0.01), (day 30 vs. baseline), (p<0.05), (day 30 vs day 1), and EF% (p<0.01), (day 30 vs baseline), (p<0.05), (day 30 vs day 1) were significantly reduced.

### Long-term upregulation of cardiac leptin mRNA and leptin synthesis in the heart did not increase plasma leptin levels

**Fig 3:**
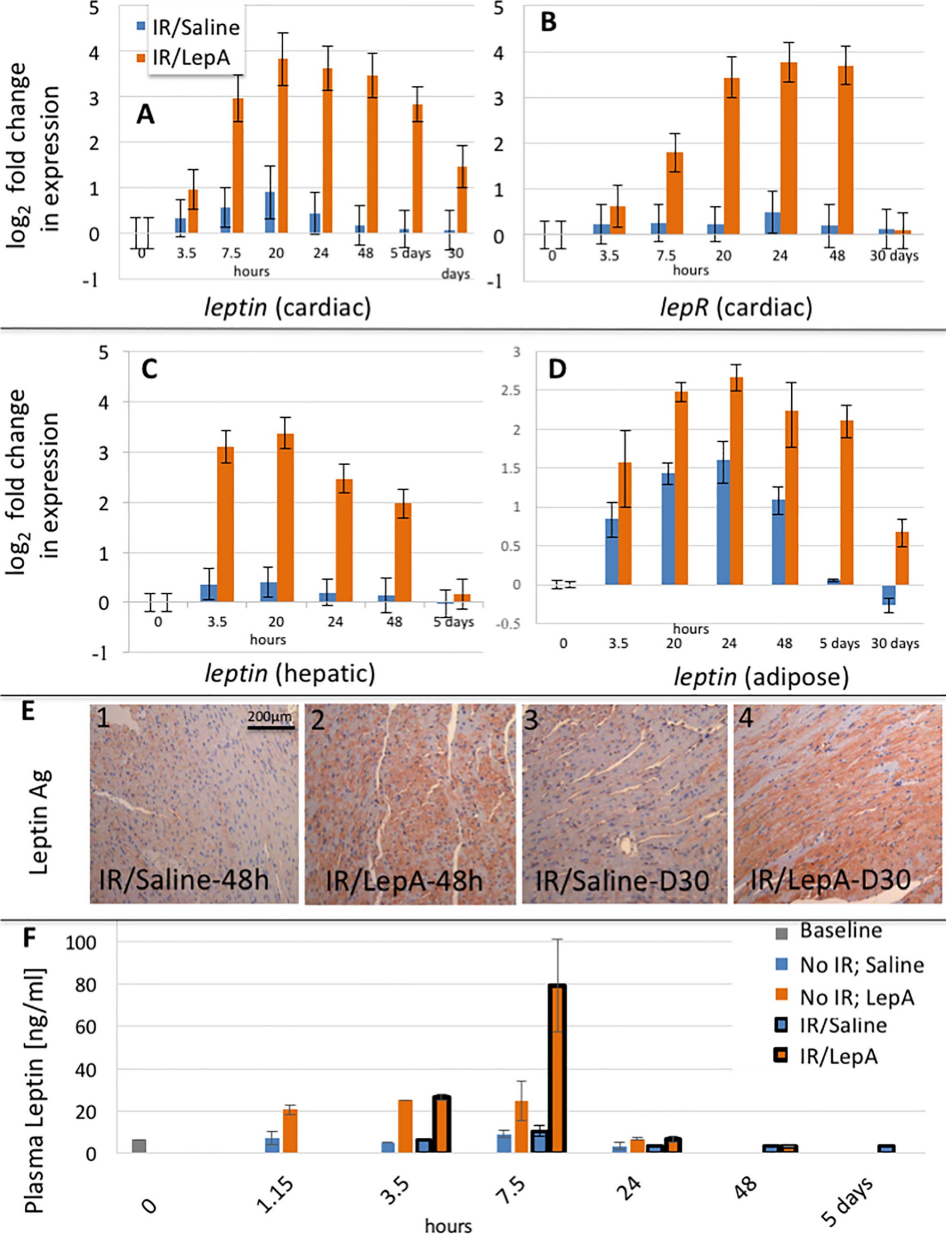
Kinetics of leptin and leptin receptor levels. (A-D): Gene expression time course analysis: *leptin* mRNA log_2_ fold change in expression in IR/saline and IR/LepA treated mice in the heart (A), liver (C), adipose tissue (D), Cardiac leptin receptor (*lepr*) mRNA log_2_ fold change in expression in IR/saline and IR/LepA treated mice (B). (E): Immunohistochemical analysis of leptin antigen in hearts from IR/saline and IR/LepA treated mice, at 48 hours (E-1,2) and 30 days (E-3,4) postoperatively. (F): Plasma leptin level determined by ELISA (ng/ml). In one separate group mice were treated by a single IP injection of LepA or saline without IR, while other mice were subjected to IR/LepA or IR/saline treatment. Note, peak of plasma leptin at 7.5 hours in both groups, subsiding to normal levels by 24 hours. (6 samples from each group were used for leptin IHC analysis).

IR/LepA samples revealed upregulation of leptin mRNA in the heart, epididymal fat, and liver (Fig 3A, 3C and 3D). Leptin mRNA levels peaked in the heart, liver, and in adipose tissue 20, 20, and 24 hours PO, respectively. Long-term increase in leptin mRNA expression was demonstrated in liver samples up to 48 hours, while in adipose tissue and heart it extended beyond POD-30. In contrast, heart samples from IR/Saline treated mice exhibited only a mild increase in leptin mRNA 20 hours postoperatively. The transcript for leptin receptor (lepR) in hearts from IR/LepA treated mice was also augmented and peaked 24 hours postoperatively (Fig 3B). The kinetics of lepR gene expression correlated leptin mRNA expression, except for its decline earlier than POD-30. Leptin synthesis was revealed by IHC analysis for leptin performed on IR/saline and IR/LepA samples, collected 48 hours, and 30 days after surgery. Leptin antigen was identified mostly in cardiomyocytes. The extent and intensity of the leptin signal were higher in IR/LepA samples compared to IR/Saline at both time points (Figs 3E1 and 4).

**Fig 4:**
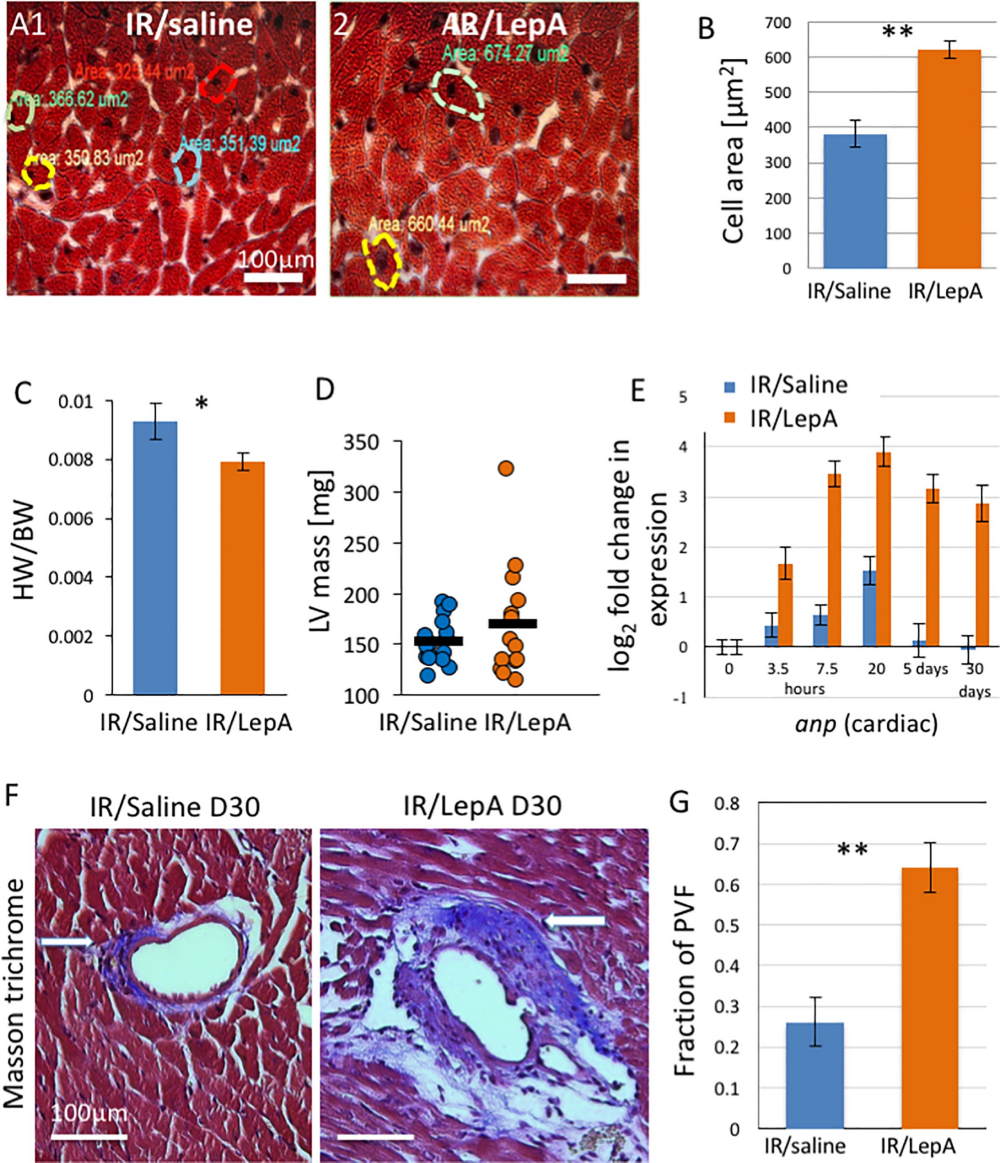
LV hypertrophy and perivascular fibrosis. (A1-2,B) Hypertrophy assessment—cardiac muscle fiber cross section area (μm^2^) in IR/saline vs IR/LepA treated mice on POD-30, using Masson trichrome stained slides, scale bar 50μm, p<0.001, two tailed t-test; (C) Heart weight to body weight ratio (HW/BW) in IR/saline vs IR/LepA treated mice, p<0.05; (D) LV mass on POD-30 in IR/saline vs IR/LepA. (E) Gene expression of *anp* mRNA log_2_ fold change in expression in IR/saline and IR/LepA treated mice in the heart; (F) Perivascular fibrosis in hearts of IR/saline vs IR/LepA treated mice on POD 30, scale bar 100μm, p<0.001, two tailed t-test (F). (6 samples from each group were assessed).

We investigated whether increased cardiac leptin gene expression and protein synthesis were reflected in systemic circulation. Plasma leptin levels were analyzed by ELISA in mice that received LepA or saline alone (without preceding IR), and in mice that were treated by IR/LepA or IR/Saline (Fig 3F). Basic leptin level (4.5 ng/ml) increased rapidly in mice receiving LepA alone, peaked to 25 ng/ml 3.5–7.5 hours after injection, returning to normal levels within 24 hours. Mice receiving IR/LepA exhibited a much higher peak of leptin level at 7.5 hours, reaching 80 ng/ml, which dropped rapidly to normal values by 24 hours. No further increase in plasma leptin level was recorded at later time points (POD 2 or 5).

### Long-term upregulation of cardiac leptin mRNA and leptin synthesis in the heart in the context of acute MI augment LV hypertrophy and perivascular fibrosis

Concomitant IR and overexpression of cardiac leptin led to cardiomyocyte hypertrophy, myocardial fibrosis, increased macrophage infiltration, and augmented capillary density as revealed on POD-30. Significant hypertrophy of cardiac muscle cells was evident in IR/LepA compared to IR/saline heart samples (p<0.001) (Fig 4A1-2 and 4B). LV hypertrophy was confirmed by comparing heart weight/body weight (HW/BW) ratio on POD-30 in the two groups (Fig 4C). Furthermore, assessment of LV mass (POD-30 in IR/LepA vs IR/saline) by echocardiography yielded no statistically significant difference for continuous variables (Fig 4D). However, when the outcome parameter was recoded using the 90^th^ percentile (cut-off value 190mg) no observations were found above cutoff 190mg in IR/saline group (n = 16), as compared to 21% above this cutoff value in IR/LepA group (n = 14). The difference was found statistically significant using 1-sided Fisher's Exact test, p = 0.045. Nevertheless, it should be noted that LV mass calculated by echocardiography in mice that sustained acute MI may provide inaccurate results attributable to large variability in location and extent of myocardial damage in the LV[24]. Therefore, echocardiography may not correlate with LV mass assessed by HW/BW ratio.

**Fig 5:**
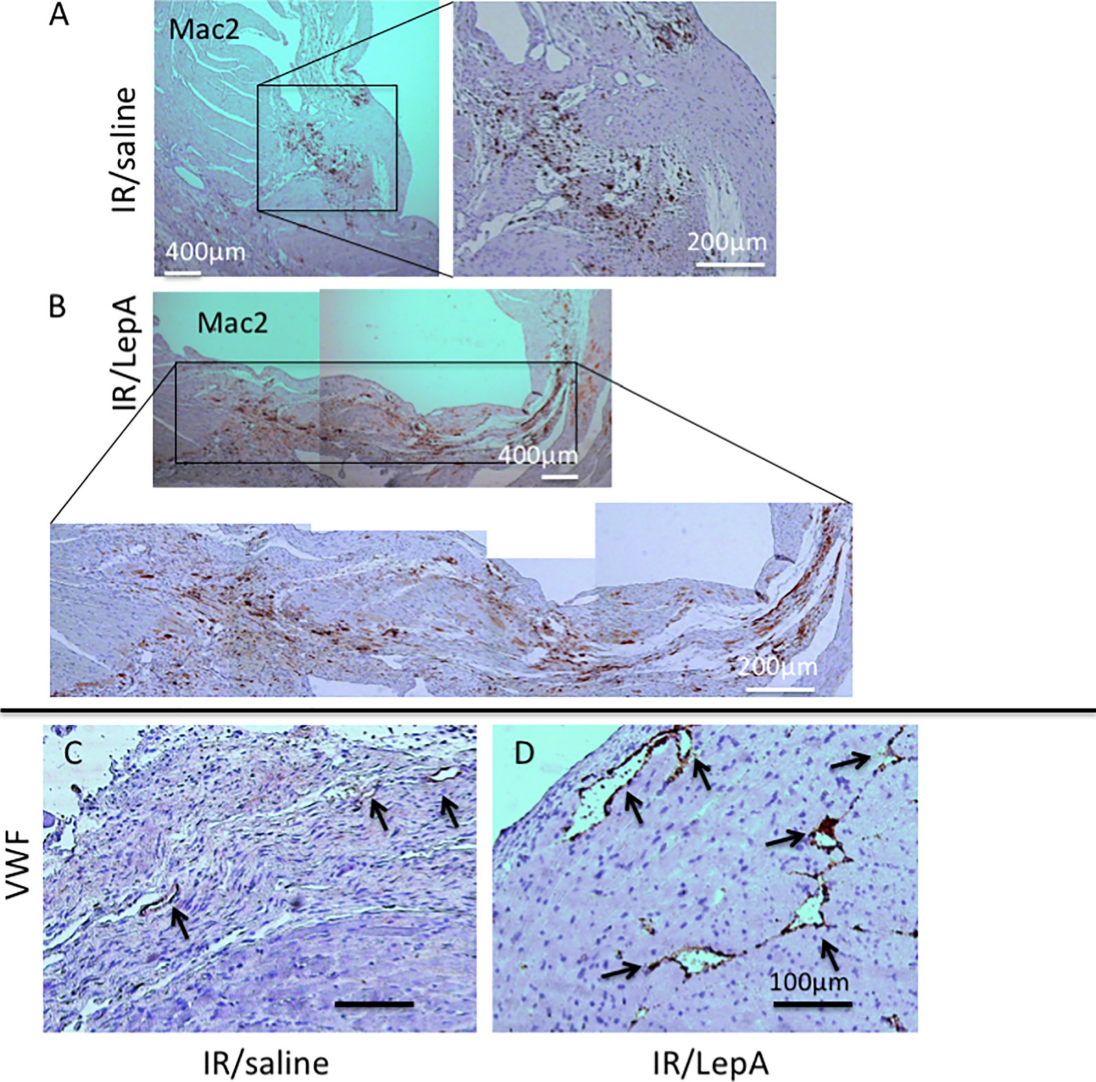
Inflammatory infiltration and angiogenesis. (A) Representative macrophage infiltration and angiogenesis in POD 30 heart samples: Macrophage infiltration in IR/saline vs (B) IR/saline treated mice demonstrated by IHC for Mac2 antigen, scale bar 400μm and 200 μm for both samples. (C,D) Angiogenesis assessment by immunohistochemistry for VWF in the same heart samples. Scale bar 100μm. (5 samples from each group were assessed).

Within the context of cardiomyocyte hypertrophy, atrial natriuretic peptide (ANP) mRNA levels were elevated in IR/LepA, and remained significantly elevated beyond POD-30 (Fig 4E). IR/saline heart samples exhibited a moderate peak at 20 hours, but there was no increased mRNA at POD 5 or 30. Perivascular fibrosis was also found on POD-30 in areas beyond infarcted myocardium in IR/LepA sample, while it was minimal or absent in IR/saline samples (p<0.001)(Fig 4F and 4G). No interstitial fibrosis was diagnosed in POD-30 samples from both treatment groups. Mac2 analysis in POD 30 heart samples demonstrated a higher density of macrophage infiltration in IR/LepA (Fig 5A and 5B), as well as augmented angiogenesis (Fig 5C and 5D).

### Cardiac TGFb1 transcript is upregulated and Smad2 pathway is activated, while STAT3 gene expression and p-STAT3 antigen are decreased in IR/LepA treated mice

**Fig 6:**
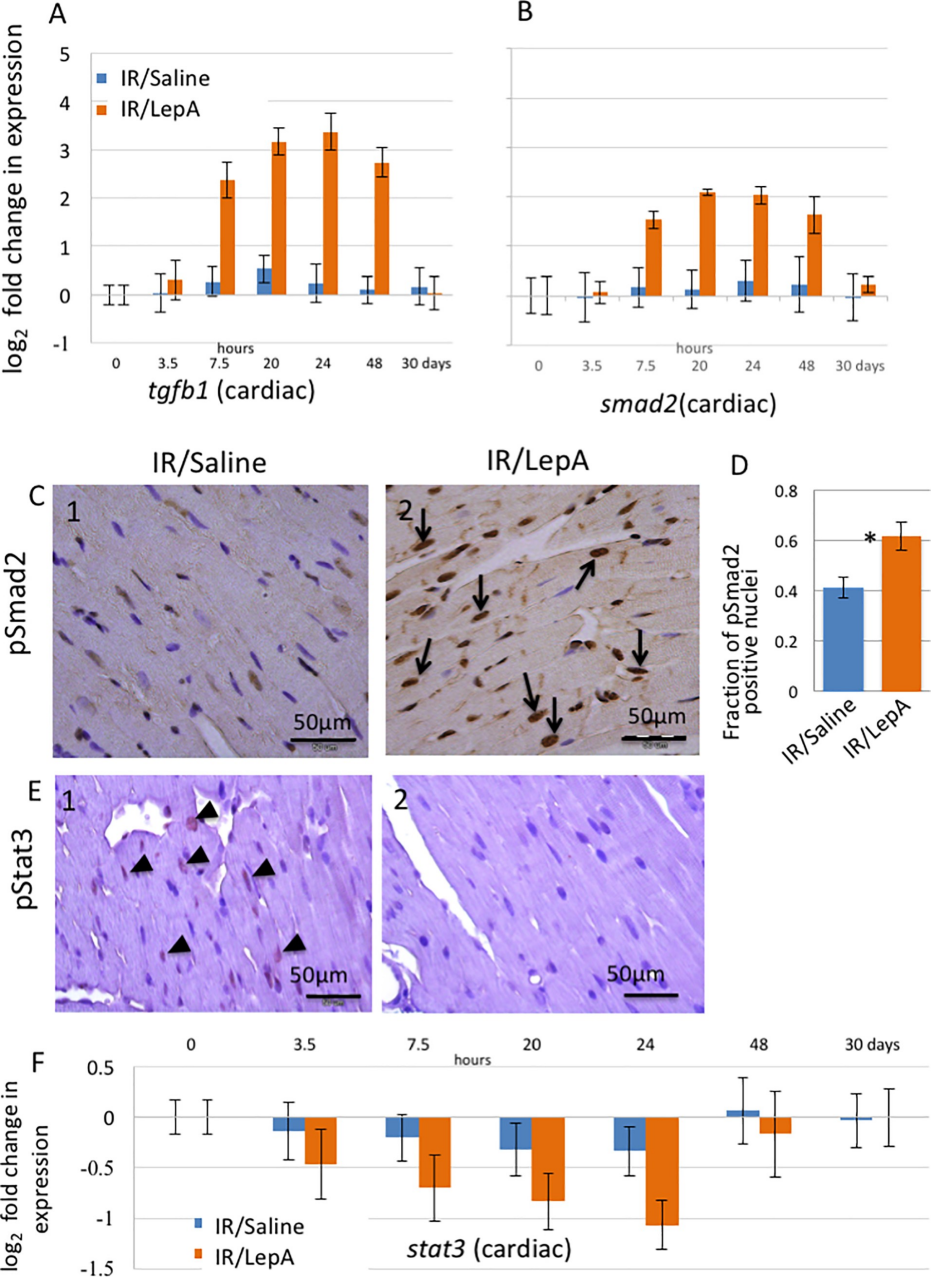
Analysis of cardiac signaling pathways. Gene expression time course analysis of *tgfb1* (A) and *smad2* (B) log_2_ fold change in expression in IR/saline vs IR/LepA treated mice in the heart. IHC analysis of phosphorylated Smad2 in heart samples from POD-30 collected from IR/saline (C1) and IR/LepA (C2) treated mice. Scale bar 50μm; p<0.001, two tailed t-test (D). Arrows point at positive nuclear signal, stained brown. Time course analysis for STAT3 mRNA, log_2_ fold change in expression in IR/saline and IR/LepA POD-30 cardiac samples (F). IHC analysis of phosphorylated STAT3 in heart samples from POD-30 collected from IR/saline (E1) and IR/LepA (E2) treated mice. Scale bar 50μm. Arrowheads point at positive nuclear signal, stained light brown. (5 samples from each group were used for IHC analysis).

Cardiac TGFb1 mRNA in IR/LepA treated mice was markedly upregulated. Its kinetics correlated with leptin gene expression during the first PO days, but declined on POD-30 (Fig 6A). IR/saline treated mice exhibited only a mild increase of cardiac TGFb1 mRNA at 20 hours PO.

Smad2 transcript correlated with TGFb1 mRNA levels in IR/LepA POD-30 samples (Fig 6B). To investigate Smad2 pathways, IHC analysis of phosphorylated Smad2 revealed multiple nuclei positive for P-Smad2 signal in IR/LepA as well as IR/saline samples, at 48 hours and 30 days, postoperatively. However, P-Smad2 signal was more prevalent in IR/LepA samples (p<0.05)(Fig 6C1-2 and 6D).

To gain further mechanistic molecular insight, we investigated STAT3 mRNA, which is known to provide cardioprotection in the context of acute MI. IR/LepA treated mice exhibited an early down-regulation of STAT3 transcript, reaching its negative peak at 24 hours PO, then normalized after 48 hours (Fig 6F). This decline in STAT3 expression was reflected in p-STAT3 synthesis as revealed by IHC analysis. We identified infrequently clusters of cells with positive nuclear signal in IR/saline (Fig 6E1), while IR/LepA samples were mostly devoid of positive signal (Fig 6E2).

## Discussion

In the current study, we show that augmented cardiac leptin expression in the context of IR drives excessive myocardial remodeling and HF. We also report that although leptin was overexpressed in the myocardium, plasma leptin levels remained within normal range, and long-term hyperleptinemia was not detected.

The role of leptin in acute MI and subsequent revascularization *in vivo* had not been fully investigated. Previous studies have attributed cardioprotective properties to cardiac leptin, presumably for preservation of myocardial function. However, many of these models may not be allowed for clinical translation, as studies utilized leptin or leptin receptor deficient animals, which necessarily cause profound systemic perturbations associated with chronic disruption of leptin pathways. Also, experiments in which exogenous leptin was infused could hardy simulate real-life pathophysiology of hyperleptinemia, since extrinsic leptin uploaded into the circulation is likely to remain dissociated from leptin expression at the tissue level. To avoid these substantial limitations, we took advantage of a strategy to stimulate visceral leptin synthesis by a brief disruption of leptin pathways. LepA, which was administered in our model as a single IP bolus injection, briefly disrupted the leptin / leptin receptor pathway before being washed out. LepA bolus did not induce detectable systemic morbidity, nor led to weight gain in IR/LepA treated mice. Additionally, the kinetics of plasma leptin as determined by ELISA reflected newly synthesized leptin rather than the administered LepA itself[25].

A single LepA IP injection most likely activated a paracrine pathway, which drove long-term endogenous leptin overexpression in multiple organs and tissues, including the heart, adipose, and liver. Leptin mRNA expression in the myocardium was significantly amplified when associated with cardiac IR injury. Myocardial tissue exhibited strong and sustainable augmentation of leptin gene expression, and this trend persisted beyond POD 30, demonstrating that locally synthesized leptin is available to fuel paracrine and autocrine pathways. Notably, leptin receptor transcript also presented a long-term upregulation, which correlated with leptin pattern of induction. Adipose tissue exhibited a long-term upregulation of leptin, demonstrating that stimulated adipocytes are a major source of newly synthesized leptin. The liver presented a moderate short-term upregulation of leptin mRNA, suggesting its responsiveness to major disruption of the leptin pathway. Unexpectedly, despite an intense and long-term augmentation in cardiac and adipose tissue leptin mRNA level and subsequent synthesis, IR/LepA mice did not present a corresponding long-term elevation in plasma leptin levels. This observation undermines the notion that plasma leptin may reflect an occult CV pathology.

Mice subjected to IR and cardiac leptin overexpression exhibited LV hypertrophy, dilation, and perivascular fibrosis. Myocardial remodeling and severe systolic dysfunction were associated with upregulated cardiac TGFβ. This finding is consistent with multiple data from previous studies in murine models of acute MI. TGFβ has been shown to mediate AngII-driven cardiomyocyte hypertrophy, promoting post-MI cardiac remodeling, apoptosis, and fibrosis[26]. Augmented AngII in the circulation and myocardilum, as well as ET-1, drives LV remodeling via TGFβ activation. Cardiomyocyte leptin, which activates MMPs and increases ROS production, promotes TGFβ synthesis and activation *via* paracrine pathways[27]. Within a similar context, we have recently demonstrated that locally synthesized leptin induced TGFβ expression in aortic wall SMCs, which in turn drove local medial degeneration and aneurysm formation in ApoE^-/-^ mice[28]. Furthermore, TGFβ mediates apoptosis and promotes myocardial fibrosis through epithelial and endothelial cell trans-differentiation to mesenchymal cells, and enhances ECM synthesis through Smad3 dependent pathways[29]. Our data corroborated these findings as we found increased Smad2 mRNA and augmented P-smad2 in IR/LepA treated mice, both indicative of TGFβ involvement in myocardial remodeling. Subsequently, IR/LepA treated mice exhibited cardiomyocyte hypertrophy, increased fibrosis, angiogenesis, and elevated inflammatory response. Both angiogenesis and increased inflammation could be attributed to excessive leptin availability.

ANP is a major cardioprotective protein, which is synthesized by cardiac myocytes in acute MI. It counteracts hypertrophy and fibrosis, reducing the extent of post MI remodeling and infarct size[30]. ANP preserves cardiac structure and function through a variety of actions, such as inhibition of AngII and ET-1, inhibition of apoptosis, reduced activation of NF-kB, and inhibited secretion of inflammatory mediators like TNFα and IL-1β. Post-MI myocardial fibrosis is antagonized by ANP, which inhibits collagen synthesis and fibroblast proliferation[31]. Our results reveal long-term upregulation of ANP gene expression in the heart of IR/LepA treated mice. ANP induction is known to be driven by multiple factors, including augmented TGFβ in cardiomyocytes, and also by AngII, ET-1, fibroblast growth factor, insulin growth factor-1, physical stress, and anoxic environment[32], most of which were present in our model. We demonstrated that, despite significant long-term overexpression of cardiac ANP, IR/LepA treated mice present with hypertrophy and fibrosis, resulting in significant LV dysfunction. This implies that abundance of cardiac leptin in the context of acute MI and reperfusion overweighs the impact of a cardioprotective factors like ANP, emphasizing the deleterious role of excessive cardiac leptin in post-MI remodeling and subsequently HF.

The signal transducer and activator of transcription 3 (STAT3) is essential for cardiac homeostasis, and its deregulation causes adverse remodeling after MI[33]. STAT3 counteracts oxidative stress in acute myocardial ischemia, exerting cardioprotective activity *via* preservation of mitochondrial integrity and modulation of transcriptional responses[34]. Results from an *ex vivo* IR rat model suggest that leptin activates STAT3[35]. However, we found that overexpressed cardiac leptin in the context of IR significantly reduced STAT3 expression at 24 hours PO, and reduced p-STAT3 below detectable level on POD-30. This may be held accountable for hampered cardioprotection in IR/LepA treated mice. Furthermore, it has been previously demonstrated that deletion of STAT3 in myocardial cells renders the heart susceptible to inflammatory damage[36]. It is likely that downregulated STAT3 in IR/LepA hearts, coinciding with leptin-induced increased macrophage density, could have contributed to excessive myocardial damage. Additionally, although decreased STAT3 signaling is associated with reduced capillary growth[37], enhanced angiogenesis, as encountered in POD-30 IR/LepA heart samples, could have been attributed to excessive leptin availability.

The severity of myocardial ischemia and the magnitude of reperfusion injury relate most likely to “time to reperfusion,” which in turn correlates with cardiac leptin synthesis. Therefore, delayed revascularization sets the stage for augmented post-MI remodeling and severe HF. Furthermore, our mouse model may also simulate clinical scenarios of patients, who experience acute MI and reperfusion coinciding with leptin overexpression associated with a preexisting inflammatory state. Grave consequences attributed to this coupling have been observed in patients suffering from inflammatory bowel disease[38,39] or rheumatoid arthritis[40], who exhibit disproportional degree of post-MI HF. We further speculate that our model may be analogous to acute MI coinciding with an inflammatory response that is incited by acute infection and sepsis (soft tissue infection, urinary tract infection, upper respiratory infection, or periodontal infection). Thereby, severe infection may render patients vulnerable to serious cardiovascular complications[41].

Our results imply that worse outcome of post-MI cardiac dysfunction may be attributed to excessive cardiac leptin synthesis. It is conceivable that effective reduction of post-MI HF in humans may be achieved through selective therapy to counteract leptin activity exclusively in the traumatized myocardium tissue, synchronized with reperfusion.

### Study limitations

While discussing clinical scenarios, which are likely analogous to our mouse model we made a few assumptions. This applies to a positive correlation between post-MI time-to-reperfusion and the extent of *in situ* cardiac expression. Also, based on multiple indirect clinical data, we have suggested that active inflammation, which coincides with acute MI and reperfusion, may underlie preexisting state of cardiac leptin oversynthesis. Both unique clinical setups can’t be directly investigated since sampling human myocardial tissue during acute MI is unethical, and therefore unobtainable. Thus, our assumptions will have to be validated by dedicated animal models.

In summary, we demonstrated that overexpressed cardiac leptin, coinciding with myocardial ischemia and reperfusion, potentiates myocardial remodeling. This occurs *via* cardiomyocyte hypertrophy and fibrosis, and leads to augmented post-MI heart failure. Our mouse model may be simulating a clinical scenario of acute MI with delayed reperfusion, or myocardial IR in patients that suffer from active inflammation, that is associated with endogenous leptin induction. We also confirmed that long-term upregulation of visceral leptin synthesis, at least in cardiomyocytes, is not necessarily translated into elevated plasma leptin levels. Therefore, measurable plasma leptin is an unreliable marker of overexpressed cardiac leptin and does not reflect accurately the presence of a leptin-related CV risk factor. Since we have shown that the magnitude of post-MI heart failure correlates with cardiac leptin expression level, therapeutic strategy to mitigate post-MI HF should target *in situ* cardiac leptin activity via local approach.

### Clinical implications and significance

This study highlights the deleterious effects of overexpressed cardiac leptin in the context of acute MI and reperfusion. Myocardial IR coinciding with overexpression of cardiac leptin results in excessive remodeling and potentiated myocardial dysfunction. Our model may simulate a common clinical scenario of acute MI with delayed reperfusion. It may also mimic myocardial ischemia, which occurs in patients that coincidentally suffer from active systemic inflammation or severe infection, associated with leptin induction, thereby rendering patients vulnerable to severe CV consequences. Our data demonstrate the uncoupling between cardiac leptin expression and leptin levels in the plasma, thus undermines the dogma that leptin level in peripheral blood is a reliable marker for the presence of active CV disease. Clinical implementation of strategies related to our findings suggests that rapid post-MI emergent therapy to antagonize local cardiac leptin activity may prove efficient to reduce the extent of subsequent post-MI HF, regardless of leptin plasma levels.
